# Comparison of Rheumatoid Arthritis Patients’ 2-Year Infliximab, Abatacept, and Tocilizumab Persistence Rates

**DOI:** 10.3390/jcm11205978

**Published:** 2022-10-11

**Authors:** Laetitia Diep, Vincent Barbier, Marie Doussière, Estelle Touboul, Claire Jesson, Valentine Deprez, Jean-Marc Sobhy-Danial, Patrice Fardellone, Vincent Goëb

**Affiliations:** 1 Department of Rheumatology, Amiens University Hospital, 80000 Amiens, France; 2 Department of Physical Medicine and Rehabilitation Center, Amiens University Hospital, 80000 Amiens, France

**Keywords:** rheumatoid arthritis, infliximab, abatacept, tocilizumab, persistence, retention

## Abstract

Background: Drug persistence reflects an agent’s efficacy and safety in routine practice. This study was undertaken to compare the 2-year persistence rates of three biologic disease-modifying antirheumatic drugs (bDMARDs) used to treat rheumatoid arthritis (RA) and to describe their efficacy and safety profiles. Methods: This retrospective, observational, single-center study included RA patients who had received at least one intravenous dose of infliximab, abatacept, and/or tocilizumab. Two-year drug persistence was estimated using the Kaplan–Meier method. Efficacy profiles were assessed as changes of Disease-Activity Score-28 (DAS28)-based EULAR-criteria responses. Results: The infliximab, abatacept, and tocilizumab groups included 40, 72, and 93 patients, respectively. Their respective 2-year persistence rates were similar: 55.0%, 45.8%, and 62.4%. Tocilizumab recipients benefited from greater improvement than those given infliximab (*p* = 0.0005) or abatacept (*p* < 0.0001). For all groups combined, 93.1% of patients obtained good or moderate EULAR responses. Conclusions: Even if this retrospective work includes different biases (lack of data, recruitment bias, etc.), it highlights that the 2-year persistence rates for infliximab, abatacept, and tocilizumab in daily practice did not differ significantly, thereby confirming the long-term efficacies of these three bDMARDs. However, tocilizumab was associated with more significant DAS28 improvement at 2 years than infliximab and abatacept.

## 1. Introduction

Rheumatoid arthritis (RA) is the most common chronic inflammatory rheumatic disease of adults in France [1]. Major progress has been made in the therapeutic management of RA over the last few decades, particularly since the advent of biologic disease-modifying antirheumatic drugs (bDMARDs) that have dramatically improved RA prognoses. Among them, tumor necrosis factor-alpha (TNF-alpha) antagonists, e.g., infliximab [2], were the first used to treat RA. Many other therapeutic agents subsequently emerged, including anti-interleukin-6 (IL6; tocilizumab) [3] and anti-cytotoxic T-lymphocyte antigen-4 (CTLA-4; abatacept) [4], which are now commonly prescribed. 

Drug persistence is a good reflection of an agent’s real-life effectiveness and safety [5]. Comparative treatment-retention studies have existed for many years, particularly in the field of inflammatory diseases but, to our knowledge, few data are available comparing the persistence rates of those three intravenous (IV) bDMARDs, which exhibit different mechanisms of action.

The primary objective of this study was to compare the 2-year drug-persistence rates of IV infliximab, abatacept, and tocilizumab of RA patients followed at a French university hospital. The secondary objectives were to analyze these three bDMARDs’ efficacies in daily practice and describe their safety profiles by collecting reasons for their discontinuation.

## 2. Materials and Methods

### 2.1. Patient Selection

This observational, retrospective, single-center study was conducted in our rheumatology department on patients who, between January 2005 and January 2018, received at least one IV infusion of infliximab, abatacept, or tocilizumab—alone or in combination with another DMARD—to treat moderate-to-severe active RA.

Patient inclusion criteria were: age 18 years or older; with a confirmed RA diagnosis meeting American College of Rheumatology/European Alliance of Associations for Rheumatology (ACR/EULAR) 2010 criteria; active RA defined as having an elevated Disease Activity Score (DAS)—DAS28 and/or DAS28-C-reactive protein (CRP)—>2.6; having received at least one IV infusion of infliximab, abatacept, and/or tocilizumab; available follow-up lasting at least 6 months; and with no objection to the use of their data.

We did not include patients unable to provide written consent or who expressed opposition to inclusion.

### 2.2. Data Recorded

For each patient, the following information collected retrospectively from electronic medical records as part of routine clinical practice was analyzed: demographic features; medical history; RA characteristics; clinical signs and symptoms; laboratory findings; DAS28 and DAS28-CRP; and any combined and/or concomitant therapies.

Drug-persistence was defined as continued bDMARD administration after 2 years because of RA remission (DAS28 < 2.6), low disease activity (=2.6 DAS28 ≤ 3.2), or DAS28 improvement corresponding to good or moderate DAS28-based EULAR-criteria responses (henceforth, EULAR responses). 

Efficacy, assessed as a DAS28 diminution, was described as the percentages of good, moderate, and non-responders, according to EULAR criteria at month (M) 12 and M24. Safety profiles were analyzed using the numbers and reasons for the discontinuation of these three bDMARDs.

### 2.3. Statistical Analyses

Categorical variables are expressed as numbers (percentages). Quantitative variables are expressed as means ± standard deviations, when the distribution was Gaussian, or as medians (range) for non-Gaussian distribution. Drug-persistence probabilities were estimated with the Kaplan–Meier method and compared using the log-rank test. Censored variables corresponding to patients who pursued bDMARD treatment after 2 years were included in the analysis. Statistical analyses were computed with SPSS Statistics software 28.0.0 (IBM Company, Chicago, IL, USA); an alpha risk of 0.05 defined significance. 

### 2.4. Ethics

A declaration of conformity to a reference methodology was made to the National Commission on Informatics and Freedoms (CNIL), and the project research protocol was read and approved by the Clinical Research and Innovation Department of the Amiens–Picardie University Hospital (registration number PI2021_843_0068).

## 3. Results

### 3.1. Demographics

Among the 334 patients retrieved from the database, 205 met the inclusion criteria: 40 received infliximab, 72 abatacept, and 93 tocilizumab. Their demographic characteristics are summarized in Table 1.

At bDMARDs onset (baseline), our three groups were comparable, except for disease duration, which was significantly longer for abatacept recipients (*p* = 0.007), and abatacept and tocilizumab was significantly more likely to be started as a monotherapy, in agreement with current management recommendations. Most RA patients exhibited positive serology for rheumatoid factor (RF) and/or anti-citrullinated peptide antibodies (ACPA)). Extraarticular manifestations were found in 18.0% of the patients, mainly as rheumatoid nodules, sicca syndromes, and pulmonary disorders, e.g., interstitial lung disease or pleurisy. DAS28 were similar for the three groups and reflected strong RA activity at bDMARD initiation. The majority of patients were taking methotrexate in combination with the reference drugs, and over half of the patients—more often, the infliximab recipients—were on corticosteroids.

### 3.2. Drug-persistence Comparisons

**Figure 1:**
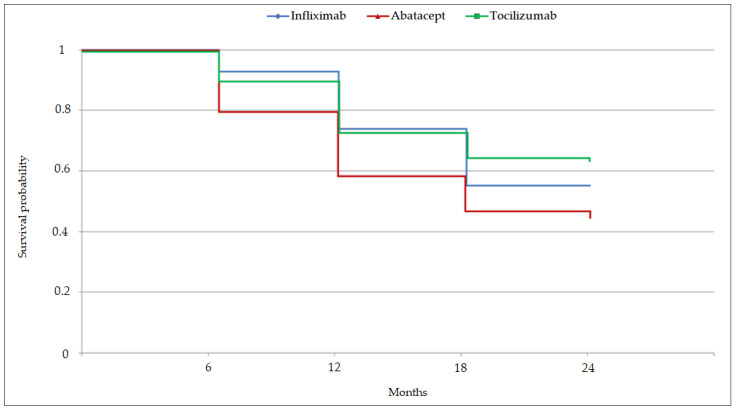
Kaplan–Meier estimations of the probability of bDMARD persistence at 2 years.

According to the Kaplan–Meier estimates (Figure 1), the probabilities of infliximab, abatacept, and tocilizumab persistence at 2 years were 55.0%, 45.8%, and 62.4%, respectively. Therapeutic maintenance did not differ significantly among the three groups (*p* = 0.064) (Table 2). However, tocilizumab retention seemed to be better during follow-up, with a mean persistence duration of 19.6 months. On the other hand, abatacept persistence lasted only a mean of 17.0 months. Among the 205 patients included in this study, 92 (44.9%) had stopped their treatment at the end of the 2-year follow-up, with the majority of discontinuations occurring during the first 12 months.

### 3.3. Effectiveness Profile

**Figure 2:**
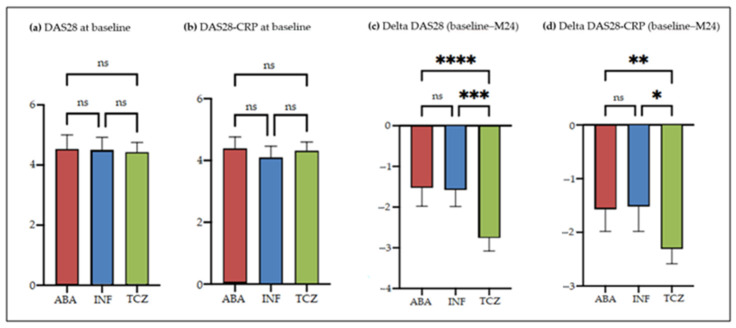
(**a**) Comparison of DAS28 at baseline; (**b**) comparison of DAS28-CRP at baseline, (**c**) DAS28 changes between baseline and M24 of treatment with infliximab (INF), abatacept (ABA), or tocilizumab (TCZ); (**d**) DAS28-CRP changes between baseline and M24 of treatment with INF, ABA, or TCZ. ns: non-significant; *: *p* = 0.012; **: *p* = 0.006; ***: *p* = 0.005; ****: *p* < 0.001.

Among patients treated with infliximab, abatacept, or tocilizumab, respective mean DAS28 (4.51 ± 0.93, 4.53 ± 1.35 and 4.43 ± 1.26) and DAS28-CRP (4.10 ± 0.82, 4.39 ± 1.07 and 4.31 ± 1.08) did not differ significantly at bDMARD onset (Figure 2a,b).

At the end of 2 years of administration, tocilizumab-treated patients still showed significant DAS28 improvement, with mean decreases of 2.76 points for DAS28 and 2.31 points for DAS28-CRP. In comparison, patients receiving infliximab or abatacept exhibited smaller activity score declines, with respective mean decreases of 1.58 and 1.52 points for DAS28, and 1.52 and 1.57 points for DAS28-CRP. The differences between these changes were statistically significant, with tocilizumab achieving greater improvement than abatacept or infliximab (Figure 2c,d). 

**Figure 3:**
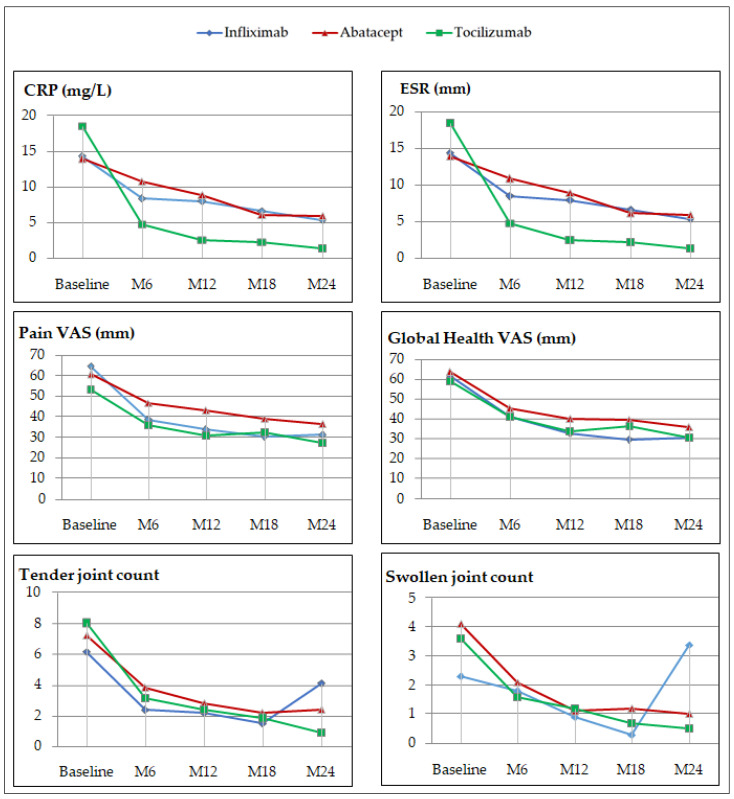
Changes in CRP and ESR levels, pain and global health VAS, and numbers of tender and swollen joints during the 2-year observation CRP: C-reactive protein; ESR: erythrocyte sedimentation rate; VAS: visual analog scale.

Biological inflammatory syndrome markers diminished in our three groups, but the declines seemed to be greater for tocilizumab recipients (Figure 3). Tocilizumab recipients benefited from rapid and considerable decreases in ESR (from 21.9 to 7.5 mm) and CRP (from 17.5 to 4.0 mg/L), with respective mean reductions of 14.4 mm and 13.5 mg/L between the baseline and M6. Those lower values were maintained between the baseline and M24, with mean decreases of 17.9 points for ESR and 16.2 points for CRP. Mean ESR and CRP levels, respectively, declined by –4.7 points and –7.6 points for infliximab recipients, and –7.6 points and –5.1 points for the abatacept group. At the end of the 2 years of treatment, ESR and CRP values were globally lower in tocilizumab recipients, with respective M 24 means of 4.0 ± 4.5 mm and 1.3 ± 1.8 mg/L.

Notably, the numbers of infliximab recipients’ tender joints and swollen joints increased sharply between M18 and M24, which might predict the onset of therapeutic escape. 

The numbers of tender or swollen joints and the presence of pain or global health VAS also decreased rapidly after of the first infusion. The improvement persisted over time, with continuous declines of these parameter values during the first 6 months of treatment, followed by a relative stability between M6 and M24.

### 3.4. EULAR responses 

**Figure 4:**
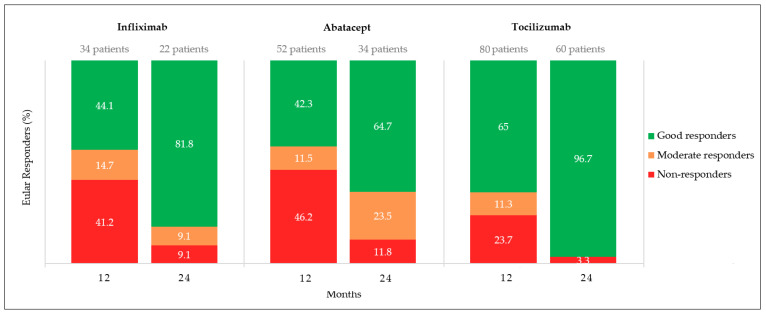
RA patient’s EULAR response rates at 12 and 24 months, according to the bDMARD being administered.

To describe bDMARD efficacy according to “responder status” using the EULAR criteria for DAS28-CRP at treatment M12 and M24 (Figure 4), patients who stopped treatment because of an adverse event were excluded. 

Overall, EULAR responses improved between M12 and M24 in all three groups, with tocilizumab recipients having the highest percentages of good responders (estimated at 65% at M12 and 96.7% at M24). 

At M12, infliximab non-responders exceeded 40%, and abatacept non-responders included >45% of recipients. 

At M24, 93.1% of all patients achieved good (84.5%) or moderate (8.6%) EULAR responses, while non-responders represented 6.9%.

### 3.5. Tolerance Profiles

**Figure 5:**
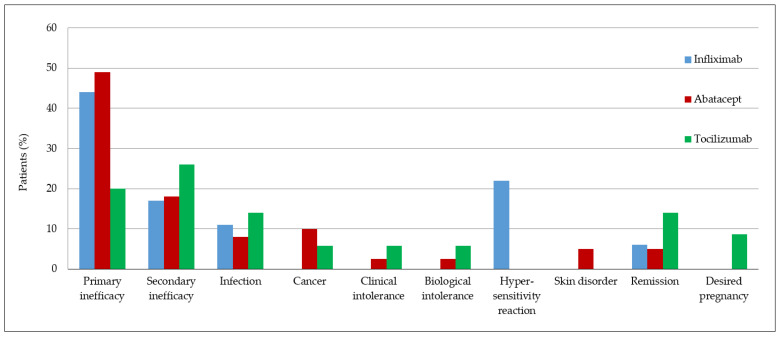
Reasons for bDMARD discontinuation.

The infliximab, abatacept, and tocilizumab tolerance profiles according to the main causes of bDMARD discontinuation during the first 2 years of treatment are shown in Figure 5. 

The most frequent reason for bDMARD discontinuation was primary inefficacy, mainly for abatacept recipients (19/39 (48.7%)), compared to 8/18 (44.4%) of the infliximab and 7/35 (20.0%) of the tocilizumab group patients. The next most common reason for discontinuation was therapeutic escape, mainly in the tocilizumab group (26.0%), compared to 18.0% of the abatacept and 17.0% of the infliximab groups.

Other less frequent reasons for stopping bDMARDs were clinical and biological intolerance, infections, cancers, hypersensitivity reactions, prolonged remissions, and the desire to become pregnant. 

Among infliximab-treated patients, seven stopped treatment: four due to allergic reactions (two bronchial hyperreactivities and two laryngeal edemas), two after developing infections (severe pneumopathy or esophageal candidiasis), and one who entered a prolonged remission.

Among abatacept-treated patients, 13 stopped their bDMARDs: four were diagnosed with cancer (gastrointestinal stromal tumor, uterine carcinoma, breast carcinoma, or lymphoma), three experienced recurrent infections (vulvar mycosis, acute otitis media, or herpes), two had skin disorders (psoriasis or rheumatoid nodules), two were in prolonged remission, one developed thrombocytopenia, and one exhibited clinical intolerance (dry cough).

Finally, 19 tocilizumab recipients discontinued treatment: five with infections (two sigmoidal diverticulitis, two ENT infections, one dental abscess), five were in prolonged remission, three wanted to become pregnant, two were diagnosed with cancers (large-cell neuroendocrine carcinoma or myelodysplastic syndrome), one became neutropenic, one became hypogammaglobulinemic, and two experienced clinical intolerance (recurrent bronchitis).

## 4. Discussion

### 4.1. Drug Persistence

In this study, 2-year infliximab, abatacept, or tocilizumab persistence rates in our RA population were comparable, with a non-significant trend towards better retention of tocilizumab and infliximab than abatacept.

Our 62.4% tocilizumab persistence rate at 2 years is comparable to that obtained from the French REGATE Registry (REGistry-RoAcTEmra) maintained by the French Society of Rheumatology and the “Club Rhumatismes et Inflammations,” which followed 1491 patients prescribed tocilizumab for refractory RA over 5 years and found a 61.3% 2-year maintenance rate [6]. However, our rate was higher than the 50% 2-year tocilizumab maintenance reported by the Swedish ARTIS Registry, based on 530 patients [7]. The Swedish patients’ overall longer-standing RA (mean 14.3 years of evolution vs. 10.9 years found herein) and more severe disease at tocilizumab initiation (mean initial DAS28 of 5.4 vs. 4.5 herein) might explain this difference.

Our abatacept persistence rate is similar to that of the international observational ACTION (AbataCepT In rOutiNe) Registry [8]; under their real-world conditions, the 2-year abatacept maintenance rate was 47.9% vs. the 45.8% found herein.

Infliximab maintenance was compared to two other TNF-alpha antagonists (etanercept and adalimumab) administered subcutaneously for RA or spondyloarthritis in a 2016 French study [9]; those authors found that RA patients had a higher retention rate for infliximab (36.88 months) than adalimumab (17.35 months) or etanercept (19.87 months). 

In 2011, Leffers et al. [10] published their comparative study on abatacept and tocilizumab efficacies and persistence rates in RA patients from the Danish Database for Biological Therapies in Rheumatology (DANBIO). As we did, they found better tocilizumab (58%) than abatacept (39%) maintenance after 96 weeks of treatment. In their French national multicenter study, Gottenberg et al. [11] demonstrated the superiority of rituximab or tocilizumab drug persistence, without failure at 2 years, over abatacept, with respective rates of 68.6%, 63.4%, and 39.3%.

Choquette et al. [12] compared the abatacept persistence rate to that of anti-TNF-alpha, including infliximab and other drugs, depending on whether they were given as a first- or second-line therapy; abatacept and anti-TNF-alpha showed similar 9-year persistence rates as the first-line bDMARD, whereas the second-line abatacept retention rate was better than that of anti-TNF-alpha. We did not distinguish among bDMARDs according to treatment line, but, on average, abatacept was given as a second-, third-, or even fourth-line treatment, while infliximab was the most frequently prescribed first-line biologic.

### 4.2. Effectiveness Profile 

Our results showed that tocilizumab provided statistically significant DAS28 improvement at 2 years, compared to infliximab and abatacept. As of bDMARD initiation, lower values of clinical parameters (numbers of tender and swollen joints, pain/disease VAS) used in daily practice were obtained. 

Patients prescribed tocilizumab had clear and rapid ESR and CRP value decreases over the first 6 months of treatment. That rapid and significant CRP decline can be explained by the pharmacodynamic role of the anti-IL6-receptor (R) [13] in inhibiting the hepatic production of inflammatory proteins during the acute phase of RA. Via that inhibitory route, tocilizumab artificially lowers CRP levels and attenuates signs of inflammation, such as fever and leukocytosis.

Tocilizumab efficacy was described previously. The Japanese ROSE study [14], a 24-week multicenter phase IIIb clinical trial, compared double-blind tocilizumab (*n* = 412) to placebo (*n* = 207) for patients whose RA had not responded adequately to conventional synthetic DMARDs (csDMARDs). Tocilizumab obtained significant early responses vs. placebo for global VAS, pain VAS, and DAS28, and CRP and ESR levels improved as early as day 7. As of week 4, mean CRP levels were significantly lower for tocilizumab recipients and remained significantly lower throughout follow-up.

In conclusion, the comparative efficacy of these three IV bDMARDs favored more marked DAS28 improvement at M24 for tocilizumab-treated patients. These results must be balanced by the fact that the rate of "non-responder" patients was greater in the infliximab and abatacept groups (50.3% and 58.0%, respectively) than in the tocilizumab group (27.0%), which implies that our data for efficacy analysis may have been small.

### 4.3. Tolerance Profile

Four patients had to stop infliximab because of an immediate allergic event, such as bronchospasm or laryngeal edema that appeared within 1 h of administration; none required resuscitative management. Neither anaphylactic shock nor delayed allergic reactions were reported. Indeed, such severe allergic reactions remain rare, as corroborated by the results of the international phase III double-blind, platelet-controlled clinical trial: among the 428 patients included, several of the 340 given infliximab developed hypotension, urticaria, or dyspnea, all non-serious [15].

Concerning the risk of infection, RA itself is a risk factor for serious infections because of its inherent systemic inflammation and the concomitant altered immunological mechanisms [16]. The infection rate is even greater when patients are on bDMARDs and systemic corticosteroids, often co-prescribed. In our study, serious and non-serious infections occurred in all three groups. Two tocilizumab recipients, with no known digestive pathology, developed uncomplicated sigmoidal diverticulitis. In fact, tocilizumab is known to carry a heightened risk of diverticulitis and gastrointestinal perforation. A more recent study compared the risks of diverticulitis and gastrointestinal perforation regarding tocilizumab, rituximab, or abatacept in 4501 RA patients from the national REGATE, AIR-PR, and ORA registries [17]. The results showed that, compared to rituximab and abatacept, tocilizumab was associated with a four-fold increased diverticulitis risk, a 3.2-fold higher gastrointestinal perforation risk, and a 4.2-fold heightened gastrointestinal perforation risk specifically associated with tocilizumab-attributed diverticulitis. It should be noted that 30% of the tocilizumab-treated patients exhibited no classical clinical manifestations of diverticulitis or perforations (absence of inflammatory syndrome or an atypical manifestation, such as an occlusive syndrome), thereby highlighting that clinicians must be particularly attentive to potential risk, alerting patients and assuring closer surveillance when prescribing this bDMARD. 

The main novelty of our study was the comparison three IV bDMARDs with different mechanisms of action (anti-TNF alpha, anti-CTLA4, and anti-IL6R). Other studies have carried out evaluations comparable to ours, but without being totally similar to it because, to our knowledge, this is the first study comparing the real-life use of these three intravenous bDMARDs in RA. Although resulting from a retrospective study, we were able to limit the loss of data by choosing patients treated intravenously, who were therefore regularly followed and evaluated in a hospital setting. Thus, we were able to collect extensive clinical and laboratory information, with relatively little missing data. Real-life long-term follow-up enabled us to obtain good representativity of the RA patients seen in routine practice. 

The retrospective design and its inherent bias constituted the limitations of our work. Our study was monocentric and conducted in a university hospital, which implies a recruitment bias. That bias may also have been favored by the initial exclusion of 69 patients who received abatacept and tocilizumab subcutaneously rather than in the form of IV infusions. The patients described here were not all naïve to bDMARDS, which can naturally have an impact on the results of the study, with the increase in treatment lines used in practice inversely correlated with the chances of a good response. The groups are small, which can also influence the results observed. Infusion delays of a few days have sometimes been observed, due to minor infectious problems encountered by patients, or to professional imperatives or personal constraints. We considered that these spacings, which were few in number and of low amplitude, were not significant enough to be taken into account in our work. The use of DAS28 is a potential source of prejudice because it is biased by its partially subjective nature (patient self-evaluation of the disease), and the multiplicity of physicians interviewing the patients and conducting physical examinations at each visit to the day hospital may have resulted in a lack of reproducibility over the 2-year observation period. 

## 5. Conclusions

Unlike randomized studies—considered the “gold standard” to establish treatment efficacy—real-life observational studies such as ours provide better representation of patients seen in daily practice, along with their long-term follow-up data. They are therefore important to evaluate a drug’s efficacy and safety. 

Our results showed comparable 2-year persistence rates of IV infliximab, abatacept, and tocilizumab to treat RA. They confirmed the good efficacies of all three bDMARDs, with tocilizumab-treated patients exhibiting better M24 DAS28 improvement. The two main reasons for discontinuation of the drugs were primary inefficacy and therapeutic failure. 

Currently, bDMARDs are widely administered subcutaneously, rather than IV, to benefit from advantages in terms of accessibility and ease of use. A real-life, comparative study similar to ours comparing these three bDMARDs injected subcutaneously could be of interest to reflect current practices.

## Figures and Tables

**Table 1:** Characteristics of the 205 RA patients and bDMARD onset.

Characteristic	Infliximab (*n* = 40)	Abatacept (*n* = 72)	Tocilizumab (*n* = 93)	*p*-value
Female sex	34 (85)	54 (75)	71 (76.3)	0.472
Age (years)	53.8 ± 15.6	58.2 ±13.0	55.6 ±13.2	0.215
Body mass index (kg/m²)	28.5 ± 6.5	27.8 ± 5.6	28.1 ± 6.5	0.855
Smokers	17 (42.5)	30 (41.7)	34 (36.6)	0.734
Disease duration (years)	8.1 ± 5.8	13.1 ± 10.7	10.9 ± 8.6	**0.007**
RF positive	25 (62.5)	47 (65.3)	61 (65.6)	0.962
ACPA positive	30 (75)	49 (68.1)	70 (75.3)	0.578
RF and ACPA negative	9 (22.5)	19 (26.4)	16 (17.2)	0.384
Erosion	28 (70)	57 (79.2)	73 (78.5)	0.527
Extra-articular manifestations	8 (20)	13 (18.1)	16 (17.2)	0.971
DAS28				
Mean	4.7 ± 1.1	4.7 ± 1.2	4.6 ± 1.3	0.987
Median	4.5 (2.8–7.6)	4.8 (2.3–8.0)	4.6 (1.5–7.5)	
DAS28-CRP				
Mean	4.4 ± 1.0	4.5 ± 1.0	4.5 ± 1.1	0.899
Median	4.2 (2.7–6.8)	4.5 (2.7–7.6)	4.5 (2.2–7.0)	
Pain VAS	61.8 ± 23.0	60.1 ± 19.8	57.4 ± 21.6	0.431
Patient global health VAS	59.8 ± 25.5	63.0 ± 19.6	60.8 ± 19.0	0.713
Tender joint count	8.3 ± 7.0	8.4 ± 7.0	9.0 ± 10.0	0.861
Swollen joint count	3.2 ± 3.2	4.1 ± 3.9	3.9 ± 4.0	0.524
ESR (mm)	26.6 ± 20.7	22.3 ± 22.2	23.3 ± 21.4	0.595
CRP (mg/L)	14.6 ± 17.6	14.0 ± 25.6	18.5 ± 25.7	0.455
Monotherapy	5 (12.5)	25 (34.7)	23 (24.7)	**0.034**
Methotrexate at baseline	28 (70)	39 (54.2)	59 (63.4)	0.224
Methotrexate dose (mg/week)	16.8 ± 3.2	13.9 ± 3.6	15.4 ± 4.1	0.785
Corticosteroids at baseline	25 (62.5)	38 (52.8)	53 (57)	0.598
Corticosteroids dose (mg/d)	9.0 ± 9.3	10.7 ± 4.6	9.9 ± 6.4	0.850
Number of prior bDMARDs	2.07 ± 1.73	3.15 ± 1.32	2.56 ± 1.28	

Values are expressed as *n* (%); mean ± SD or median (range). RF: rheumatoid factor; ACPA: anti-citrullinated peptide antibodies; DAS28: Disease Activity Score 28; CRP: C-reactive protein; ESR: erythrocyte sedimentation rate; VAS: visual analog scale; bDMARD: biologic disease-modifying antirheumatic drug.

**Table 2:** Two-year drug persistence of the three bDMARDs.

	Infliximab (*n* = 40)	Abatacept (*n* = 72)	Tocilizumab (*n* = 93)	*p*-Value
Stop/Censure	18/22	39/33	35/58	0.106
	Number of patients, *n* (%)			
6 months	37 (92.5)	57 (79.2)	82 (88.2)	
12 months	30 (75)	42 (58.3)	68 (73.1)	
18 months	22 (55)	34 (47.2)	60 (64.5)	
24 months	22 (55)	33 (45.8)	58 (62.4)	
	Mean Persistence Duration, months (CI 95%)			
	19.3 (17.5–21.2)	17.0 (15.2–18.7)	19.6 (17.7–19.6)	0.064
